# Rare phenotype: Hand preaxial polydactyly associated with *LRP6*-related tooth agenesis in humans

**DOI:** 10.1038/s41525-021-00262-0

**Published:** 2021-11-10

**Authors:** Liutao Zhang, Miao Yu, Kai Sun, Zhuangzhuang Fan, Haochen Liu, Hailan Feng, Yang Liu, Dong Han

**Affiliations:** 1 Department of Prosthodontics, Peking University School and Hospital of Stomatology & National Center of Stomatology & National Clinical Research Center for Oral Diseases & National Engineering Laboratory for Digital and Material Technology of Stomatology & Beijing Key Laboratory of Digital Stomatology & Research Center of Engineering and Technology for Computerized Ministry of Health & NMPA Key Laboratory for Dental Materials, Beijing, People’s Republic of China; 2 Department of Dentistry, Beijing Jishuitan Hospital, Beijing, People’s Republic of China; 3 Department of Dentistry, Beijing Hospital, Beijing, People’s Republic of China

**Keywords:** Disease genetics, Dental diseases

## Abstract

Low-density lipoprotein receptor-related protein 6 (*LRP6*) is a pathogenic gene of selective tooth agenesis-7 (OMIM#616724). Although the malformation of the digits and fore- and hindlimbs has been reported in *Lrp6-*deficient mice, it has been rarely discovered in humans with *LRP6* mutations. Here, we demonstrate an unreported autosomal dominant *LRP6* heterozygous mutation (c.2840 T > C;p.Met947Thr) in a tooth agenesis family with hand polydactyly, and another unreported autosomal dominant *LRP6* heterozygous mutation (c.1154 G > C;p.Arg385Pro) in a non-syndromic tooth agenesis family. Bioinformatic prediction demonstrated the deleterious effects of the mutations, and LRP6 structure changes suggested the corresponding functional impairments. Analysis on the pattern of *LRP6*-related tooth agenesis demonstrated the maxillary lateral incisor was the most affected. Our study report that *LRP6* mutation might be associated with hand preaxial polydactyly in humans, which broaden the phenotypic spectrum of *LRP6*-related disorders, and provide valuable information on the characteristics of *LRP6*-related tooth agenesis.

## Introduction

Low-density lipoprotein receptor-related protein 6 (*LRP6*, OMIM*603507, cytogenetic location: 12p13.2) is a recently discovered pathogenic gene related to tooth agenesis, which was designated as autosomal dominant tooth agenesis, selective 7 (STHAG7; OMIM#616724) in 2015^1^. The human *LRP6* gene encodes a single-pass transmembrane receptor protein consisting of an intracellular domain, a transmembrane domain, and a large extracellular domain. LRP6, together with its highly homologous subfamily member-LRP5^2^, can function as an integral co-receptor for WNTs. Animal models and clinical genetic studies have confirmed that the WNT–FZD–LRP6 trimer formed on the cell membrane can initiate β-catenin-dependent WNT signaling, and thus play an important role in the development of tooth, skeleton, and other organs^3,4^. At present, *LRP6* mutations are also believed to be involved in the pathogenesis of coronary artery disease (OMIM#610947) and are considered as risk factors for cleft lip and/or palate, hypohidrotic ectodermal dysplasia, osteoporosis, neural tube development defects, and Alzheimer’s disease^5–8^. Moreover, research evidence demonstrated that homozygous *Lrp6*-deficient mouse embryos exhibited malformed fore- and hindlimbs and loss of distal limb structures^9^. The forearm and digits were seen in *Lrp6-floxdel* mouse with full penetrance of oligodactyly and low penetrance of polydactyly^4^. Oligodactyly was occasionally observed in the mouse with a spontaneous *Lrp6* point mutation *ringelschwanz (rs)*^10^. Therefore, *LRP*6 may play an important role in prenatal limb bud and skeletal development.

Polydactyly, characterized by supernumerary digits, is the most common congenital hand deformity in human, with an estimated frequency of 0.1–0.14%^11^. Based on the radioulnar side of extra digits, hand polydactyly is categorized as preaxial (radial side), postaxial (ulnar side), and central polydactyly^12^. In recent years, the genetic basis has been studied, and a dozen genes, such as *GLI3*, *GLI1*, *SHH*, *PAPA2*, *PAPA4*, *PITX1*, *MIPOLI*, *LMBR1*, *TWIST1*, and *FGFR2*^13,14^, have been identified to be involved in the pathogenesis of hand polydactyly in humans. Interestingly, a latest clinical study reported a relevance of *LRP6* frameshift mutation to the development of split-hand/foot malformation, and suggested *LRP6* as a candidate gene for split-hand/foot malformation^15^. However, the correlation between *LRP6*-related tooth agenesis and digit malformation in human remains unclear. Therefore, more clinical evidence of *LRP6* mutations is needed to confirm this important correlation between human tooth agenesis and digit deformity. Besides that, due to the loss of information about specific tooth agenesis pattern in patients with *LRP6* mutations, the phenotypic variability of *LRP6*-associated tooth agenesis needs to be further explored.

In this study, we performed whole-exome sequencing (WES) and clinical assessment of three families with inherited tooth agenesis to investigate the genetic causes and clinical phenotypes. Three pathogenic *LRP6* mutations were identified, including two unreported and one known mutation. Strikingly, a typical phenotype of hand preaxial polydactyly was discovered in members of a family with *LRP6* mutation, which suggested a genetic correlation between *LRP6*-related tooth agenesis and hand polydactyly. Furthermore, to analyze the susceptibility to tooth position caused by *LRP6* mutations, we summarized the tooth agenesis positions attributed to *LRP6* mutations in seven patients among our three pedigrees and 29 previously reported cases, and thus obtained the characteristics of the *LRP6*-related tooth agenesis pattern.

## Results

### Family #704

**Fig. 1:**
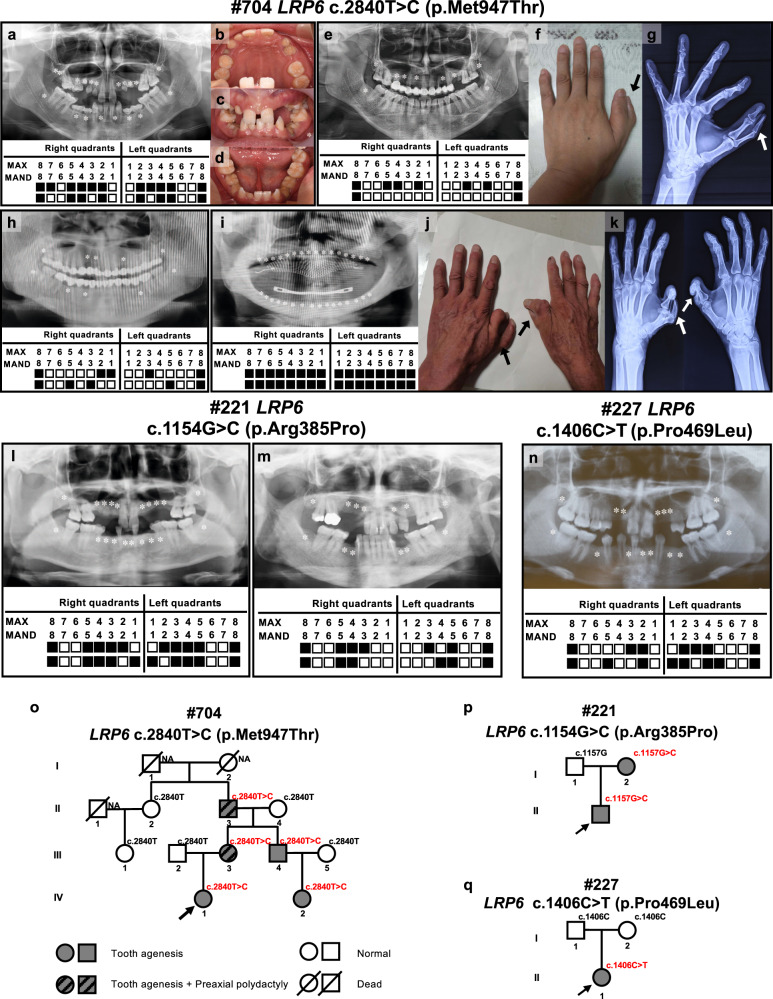
Clinical features and pedigree of families with tooth agenesis caused by *LRP6* mutations. **a–d** Panoramic radiograph, tooth agenesis schematic, and intraoral photographs of #704 proband (IV:1) with 15 congenital permanent tooth agenesis. **e** Panoramic radiograph and tooth agenesis schematic of #704 proband’s mother (III:3) with 5 congenital permanent tooth agenesis. **f**, **g** Left-hand photograph and X-ray of III:3 showed unilateral preaxial polydactyly (arrow). **h** Panoramic radiograph and tooth agenesis schematic of #704 proband’s uncle (III:4) with 6 congenital permanent tooth agenesis. **i** Panoramic radiograph and tooth agenesis schematic of #704 proband’s grandfather (II:3) with congenital missing permanent teeth. **j**, **k** The hand photograph and X-ray of II:3 showed bilateral preaxial polydactyly (arrow). **l**, **m** Panoramic radiographs, and tooth agenesis schematics of #221 family, including the proband (II:1) with 16 congenital permanent tooth agenesis, and his mother (I:2) with 9 congenital permanent tooth agenesis. **n** Panoramic radiographs and tooth agenesis schematics of #227 II:1 (proband) with 16 congenital permanent tooth agenesis. Asterisks and solid squares indicate the missing permanent tooth position. The written informed consents for publication of their photos were obtained from all the participants. **o–q** Heterozygous *LRP6* missense mutation (c.2840 T > C;p.Met947Thr), heterozygous *LRP6* missense mutation (c.1154 G > C;p.Arg385Pro), and heterozygous *LRP6* missense mutation (c.1406 C > T;p.Pro469Leu) were identified in families #704, #221, and #227, respectively. Max, maxillary; Mand, mandibular. Black arrowheads indicate the probands. NA, not available. Gray circles/squares represent patients with tooth agenesis. Gray circles/squares with black lines represent patients with tooth agenesis and preaxial polydactyly. White circles/squares represent normal relatives. White circles/squares with a long diagonal indicate deceased relatives.

In this family, a 16-year-old female proband (IV:1) presented with congenital agenesis of 15 permanent teeth (the third molars were excluded), and had shovel-shaped permanent incisors and retained deciduous teeth (Fig. 1a–d). Family investigation showed that her mother (III:3) exhibited four congenitally missing permanent teeth (Fig. 1e), and her uncle (mother’s little brother, III:4) and his daughter (IV:2) exhibited six and two congenitally missing permanent teeth, respectively (Fig. 1h and Supplementary Table 1). Her grandfather (mother’s father, II:3) was also a patient of tooth agenesis; however, in his case the affected tooth sites were untraceable due to current edentulous dentition (Fig. 1i). None of the patients with tooth agenesis from this family had dysplasia of the ectoderm or other organs, except for III:3 and II:3. Interestingly, through clinical and X-ray examinations we found that III:3 and II:3 exhibited unilateral (Fig. 1f, g) or bilateral (Fig. 1j, k) preaxial polydactyly of the hand thumbs, respectively. The defects of the left-hand thumb of III:3 and right-hand thumb of II:3 were duplicated proximal phalanx, while the left-hand thumb of II:3 manifested as bifid metacarpal. According to the Wassel classification of radial polydactyly^12^, the left hand of III:3#704 (the proband’s mother) belonged to Type IV hand polydactyly, while the left hand of II:3#704 (the proband’s grandfather) was Type V hand polydactyly and his right hand was Type IV hand polydactyly. WES and Sanger sequencing revealed an unreported heterozygous missense mutation in exon 13 of the *LRP6* (c.2840 T > C;p.Met947Thr) in three family members with tooth agenesis (IV:1, III:4, and IV:2) and two family members with tooth agenesis accompanied by hand polydactyly (III:3 and II:3) (Fig. 1o and Supplementary Fig. 1a). Importantly, we did not find any other pathogenic mutations in 586 orodental-related genes and 25 polydactyly/syndactyly related genes^11,16^, except for *LRP6* (for details see Supplementary Tables 2–4 and the online WES data, PRJNA758560). Familial co-segregation analysis showed that c.2840 T > C (p.Met947Thr) was not detected in the other unaffected relatives (Fig. 1o and Supplementary Fig. 1a), thus confirming that the inheritance of tooth agenesis in this family was dominant.

### Family #221

The proband (II:1) of family#221 was a 31-year-old man with congenital loss of 16 permanent teeth and 2 retained deciduous teeth (Fig. 1l and Supplementary Table 1). His facial features, hair, skin, and hands were normal. His mother also presented with 9 permanent teeth agenesis, but her systemic organs were normal (Fig. 1m and Supplementary Table 1). No clinical symptoms were observed in his father. An unreported heterozygous missense mutation (c.1154 G > C; p.Arg385Pro) located in exon 6 of the *LRP6* was detected in the proband and his mother by WES screening and Sanger sequencing (Supplementary Fig. 1b). This mutation was not detected in his asymptomatic father, suggesting that the proband’s *LRP6* mutation (c.1154 G > C;p.Arg385Pro) was inherited from his mother (Fig. 1p and Supplementary Fig. 1b).

### Family #227

In family#227, the 17-year-old female proband (II:1) showed the absence of 11 permanent teeth, without any obvious systemic anomalies (Fig. 1n and Supplementary Table 1). No congenital tooth agenesis or other systemic anomalies were observed in the parents. A known *LRP6* heterozygous missense mutation (c.1406 C > T;p.Pro469Leu) was detected in the proband by WES and Sanger sequencing (Supplementary Fig. 1c). This mutation was not detected in asymptomatic parents (Fig. 1q and Supplementary Fig. 1c). Therefore, the proband’s *LRP6* mutation was a de novo mutation.

### LRP6 mutations and bioinformatics findings

**Fig. 2:**
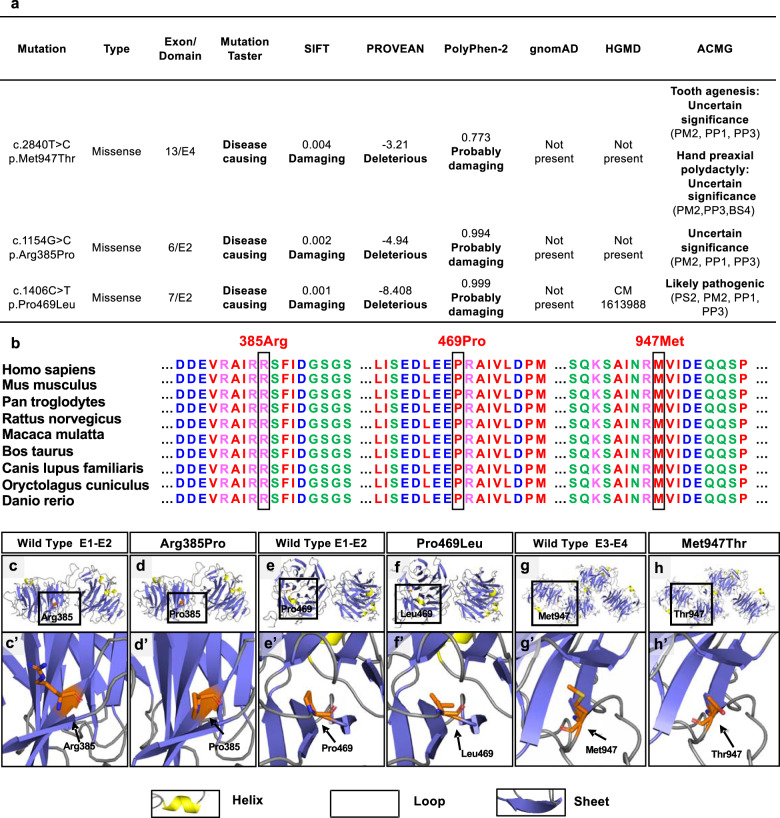
Functional impact predictions, conservation analysis of *LRP6* mutations, and structural modeling of the wild-type and mutated functional domains of LRP6 protein. **a** Functional impact prediction of two novel and one previously detected *LRP6* mutations. **b** Conservation analysis of the affected amino acids in LRP6 protein. **c–f** Structural changes in the p.Arg385Pro and p.Pro469Leu mutants compared with that in wild-type E1–E2. Dashed boxes denote the location of the Arg385 (**c**), Pro385 (**d**), Pro469 (**e**), and Leu469 residues (**f**). **g**, **h** Structural changes in the p.Met947Thr mutants compared with the wild-type E3–E4. Dashed boxes indicate the location of the Met947 (**g**) and Thr947 (**h**). **c**’–**h**’ Higher magnifications of the above boxed regions surrounding the residues. Arrows indicate the wild-type (**c**’, **e**’, **g**’) and mutated residues (**d**’, **f**’, **h**’), respectively.

To further analyze the pathogenicity of the three *LRP6* mutations, c.2840 T > C;p.Met947Thr, c.1154 G > C;p.Arg385Pro, and c.1406 C > T;p.Pro469Leu, we performed bioinformatics analysis and found that mutations c.1154 G > C;p.Arg385Pro and c.1406 C > T;p.Pro469Leu were located in the second EGF-like repeat (E2) fragment of the extracellular domain, and the mutation c.2840 T > C;p.Met947Thr was located at E4 of the extracellular domain. Although the three mutations were not present in the gnomAD database, c.1406 C > T;p.Pro469Leu was included in the Human Gene Mutation Database (HGMD). Based on online evidence from MutationTaster, Sorting Intolerant from Tolerant (SIFT), Protein Variation Effect Analyzer (PROVEAN) and polymorphism phenotyping (PolyPhen-2), the three mutations were damaging (Fig. 2a). According to the standards of 2015 American College of Medical Genetics and Genomics (ACMG), the mutations (c.2840 T > C;p.Met947Thr and c.1154 G > C;p.Arg385Pro) were predicted to be uncertain significance, while the mutation (c.1406 C > T;p.Pro469Leu) was predicted to be likely pathogenic (Fig. 2a). Therefore, further molecular investigations are required to confirm the pathogenicity.

The results of evolutionary conservation revealed that the LRP6 amino acid residues Arg385, Pro469, and Met947 were highly conserved across multiple species, indicating that subtle changes in these residues may cause significant functional impacts (Fig. 2b).

Homology modeling and spatial structural analysis were conducted to compare the conformational alterations between the wild-type and mutant LRP6 proteins. The functional domains of wild-type LRP6 consisting of E1–E2 (Fig. 2c, e) and E3–E4 (Fig. 2g) were homology modeled. Spatial structural analysis revealed that the Arg385Pro mutation resulted in a significant conformational change in the β-sheets near the 385th residue, and a longer positively charged side-chain Arg (Fig. 2c-c’) was substituted with a Pro, which possessed a hydrophobic residue with a heterocyclic ring and a shorter side-chain than Arg (Fig. 2d-d’). The Pro469Leu mutation led to the residue Pro, with a longer side-chain and an aromatic ring (Fig. 2e-e’) being substituted with a shorter side-chain Leu, which might affect the interaction of the 469th residue with its surrounding residues (Fig. 2f-f’). For the Met947Thr mutation identified in family #704 with tooth agenesis and hand thumb polydactyly, a hydrophilic Thr with a longer side-chain (Fig. 2h-h’) replaced the hydrophobic Met with a thioether bond (Fig. 2g-g’), which might affect the interaction of the 947th Met residue with the aromatic nucleus, and changes in hydrophilicity might further disrupt the intermolecular hydrogen bond. Therefore, the aforementioned conformational changes in LRP6 mutants suggested that functional experiments are required to explain the possible pathogenic mechanisms.

### LRP6-related tooth agenesis pattern

**Fig. 3:**
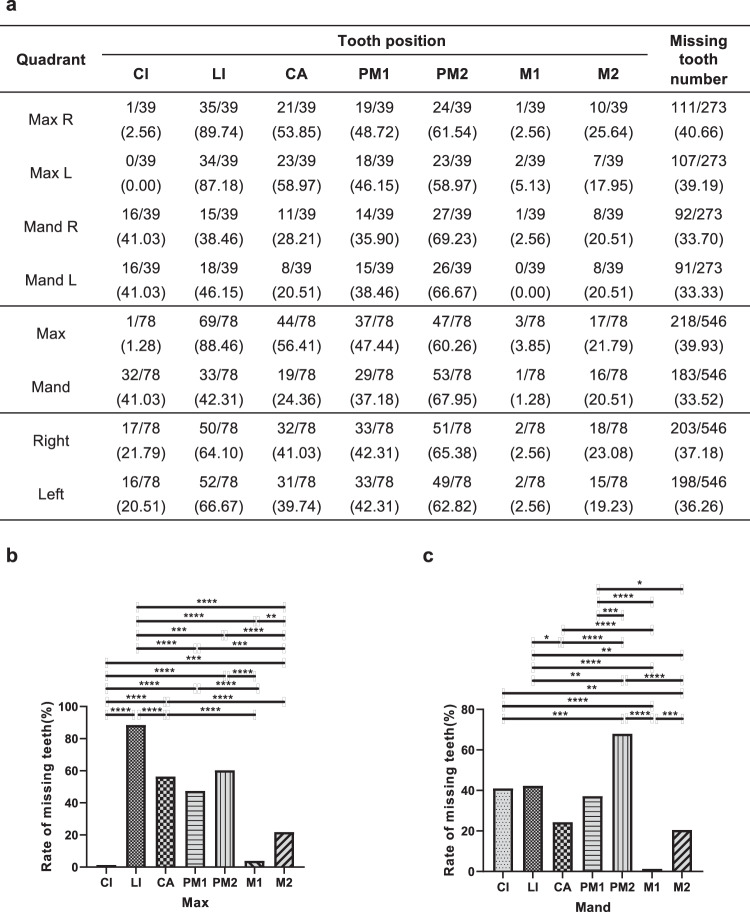
Percentage of missing tooth positions at each maxillary and mandibular dentition in all reported tooth agenesis patients with *LRP6* mutations (*n* = 39). **a** Analysis of missing tooth number 39 tooth agenesis patients with *LRP6* mutations at each tooth position of their permanent dentition (excluding the third molars). **b**, **c** Percentage of missing tooth positions at each maxillary and mandibular dentition in all tooth agenesis patients with *LRP6* mutations (*n* = 39). The numerator denotes the number of missing teeth and the denominator denotes the total number of teeth in each tooth position. The number in brackets denotes the rate of missing teeth. Max, maxillary; Mand, mandibular; CI, central incisor; LI, lateral incisor; CA, canine; PM1, first premolar; PM2, second premolar; M1, first molar; M2, second molar. Statistical significance: *P* values are: ^*^P < 0.05, ^**^P < 0.01, ^***^P < 0.001, and ^****^P < 0.0001.

Statistical analysis was used to determine the susceptible tooth positions in the permanent dentition caused by *LRP6* mutations (Supplementary Table 1). The maxillary lateral incisor (maxillary LI) was the most affected (88.46%), followed by mandibular second premolar (mandibular PM2, 67.95%) and maxillary second premolar (maxillary PM2, 60.26%), while maxillary central incisor (maxillary CI, 1.28%), mandibular first molar (mandibular M1, 1.28%), and maxillary M1 (3.85%) were the least affected (Fig. 3). The average rate of maxillary tooth agenesis (39.93%) was slightly higher than that of the mandibular dentition (33.52%), with a statistically significant difference (*P* < 0.05) (Supplementary Table 5). The agenesis rate on the left side (36.26%) was comparable to that of the right side (37.18%) (*P* > 0.05), with a distinct characteristic of bilateral symmetry agenesis in each tooth position (Supplementary Table 6).

## Discussion

To date, only 19 *LRP6* mutations have been identified in tooth agenesis in six previous studies (Supplementary Table 7)^1,17–22^. In our study, we reported a rare phenotype of hand preaxial polydactyly in a tooth agenesis family with an unreported heterozygous *LRP6* missense mutation (c.2840 T > C;p.Met947Thr). In addition, another unreported (c.1154 G > C;p.Arg385Pro) and a known heterozygous *LRP6* missense mutation (c.1406 C > T;p.Pro469Leu) were identified in two unrelated pedigrees with non-syndromic tooth agenesis. Bioinformatic analysis confirmed the deleterious effects of the mutations detected above. However, in-depth functional analyses are required to elucidate the pathogenicity of *LRP6* mutations with tooth agenesis and hand polydactyly. Nevertheless, our findings further expand the genotypic spectrum of *LRP6*-related tooth agenesis.

WNT signaling is activated when WNT proteins interact with the receptor FZD and the co-receptor LRP6/5^23^. The formation of the FZD–WNT–LRP6/5 ternary complex results in the phosphorylation of the intracellular domain of LRP6/5 and transduces the WNT signal into the nucleus^2^. In the LRP6 extracellular domain, the four E1–E4 and the three LAs are vital functional domains and are indispensable for binding with extracellular WNTs and FZD^2^. Specifically, the E1–E4 crystal structure consists of four pairs of “YWTD-type β-propeller” (E1–E4 propellers) and “EGF-like” subdomain^24^. Recent research has demonstrated that the four YWTD propeller subdomains in LRP6 share a low identity among them, suggesting functional differences among E1–E4 propellers^24^. Our spatial structural analysis showed that the E2 propeller was disrupted by p.Arg385Pro and p.Pro469Leu mutations, and the E4 propeller was disrupted by p.Met947Thr. These apparent conformational changes indicated that the side-by-side structural continuity of the four YWTD propellers, or the interaction of the EGF-like subdomain with YWTD propellers 2/4 could be seriously damaged, which may consequently affect the formation of the FZD–WNT–LRP6/5 trimer.

Strikingly, we found a rare phenotype of hand thumb polydactyly in two members of family #704, which has not been reported in patients with *LRP6*-related tooth agenesis. Similar with our finding, two recent studies reported that truncated mutations in *LRP6* led to tooth agenesis with mild malformations of the hand thumbs^17^ and clinodactyly of the fifth fingers^18^, respectively. Further studies on mouse models and molecular genetics are required to elucidate the mechanism of *LRP6* mutation cause hand polydactyly. In addition, these findings of potential correlation between tooth agenesis and hand polydactyly are consistent with the recent viewpoint that tooth agenesis is a dependent disorder that can be the regional manifestation of a systemic disease.

Limb development is an extremely complex physiological process that initiates from undifferentiated mesoderm cells encased by ectoderm^25^. The signaling centers in limb buds mutually maintain their activity and build a strong control system that integrates the pattern formation of limbs and digits^26–28^. It is well known that WNT pathway plays an important role in the etiology of tooth agenesis^19^. Meanwhile, WNT pathway controls several processes during limb and digit development and disruption of WNT signaling is involved in the pathogenesis of split-hand/foot malformation^29,30^. Besides, homeobox (*Hox*) genes, sonic hedgehog (Shh) signal transduction via Gli3 or Gli1, and its interaction with *Hox* orchestrated the correct number of digits in the vertebrate limb as well^31,32^. Based on the current studies on human cases, 25 genes are involved in the pathogenesis of polydactyly or syndactyly^11^. Through WES and Sanger sequencing, we did not detect any suspicious pathogenic mutations in the above-mentioned genes in the polydactyly family#704. Therefore, we revealed that the *LRP6* mutation may be highly correlated with human thumb polydactyly. However, whether other undiscovered genes are associated with thumb polydactyly in family#704 requires further study. In addition, patients affected with the same heterozygous *LRP6* mutation in this family exhibited different phenotypes, while patients (II:3 and III:3) showed tooth agenesis and thumb polydactyly, and patients (III:4, IV:1, and IV:2) exhibited only tooth agenesis. The phenotypic heterogeneity caused by the same *LRP6* mutation in this family may be due to incomplete penetrance or the possible effects of other genetic or epigenetic modifiers.

Tooth development is under a fine-tuning of the signaling network that regulates tooth epithelial‐mesenchymal interactions, especially the β-catenin-dependent Wnt pathway^33,34^. Our previous work clarified the dynamic expression of *Lrp6* in the dental papilla and dental epithelium, implicating its essential role in controlling epithelial-mesenchymal interactions during tooth development^19^. However, *LRP6*-related specific tooth agenesis pattern is necessary to further investigate to understand the regulatory effects of *LRP6* on each permanent tooth. We demonstrated that patients with *LRP6* mutations had a specific tooth agenesis pattern: the maxillary lateral incisor and the second premolars of both jaws were the most affected, while the maxillary central incisor and mandibular first molars were the least affected. These results strongly suggest that *LRP6* plays a critical role in the development of the human maxillary lateral incisor and the second premolars of both jaws, which will help clinical geneticists or dentists to differentiate diagnosis, target pathogenic genes in advance, and facilitate the genetic studies of tooth agenesis patients.

In conclusion, we found out hand thumb polydactyly in a tooth agenesis family with *LRP6* mutation, and further proposed a potential correlation between *LRP6* mutation and hand thumb polydactyly, which broadened the phenotypic spectrum of *LRP6*‐related disorders. The results of our study enlarged the *LRP6* mutation spectrum of tooth agenesis and demonstrated the *LRP6*-related tooth agenesis pattern. Our findings may facilitate early genetic diagnosis and perinatal counseling.

## Methods

### Family recruitment

Three unrelated families with inherited tooth agenesis were recruited from the Department of Prosthodontics at the Peking University School and Hospital of Stomatology (Beijing, China). The extracted teeth were excluded to ensure accurate statistical analysis. Detailed intraoral and radiographic examinations were performed using a prosthodontist. Hand X-rays were provided by the patients. The written informed consents from all the participants for the use of blood or saliva samples, clinical data, and publication of their photographs were obtained. All experiments were approved by the Ethics Committee of Peking University School and Hospital of Stomatology (PKUSSIRB-201736082).

### Whole-exome sequencing

Genomic DNA from each proband was extracted from peripheral blood lymphocytes using the Blood Genomic DNA Mini Kit (Cwbiotech, China), and was sent to iGeneTech (Beijing, China) for WES using the Illumina-X10 platform. To filter the detected variants, orodental-related and hand development-related genes were annotated^11,16^. Then, we excluded silent variants and missense variants with a minor allele frequency (MAF) ≥ 0.01, in East Asians. Online bioinformatics analysis software, such as SIFT, PolyPhen-2, PROVEAN, and MutationTaster were used for bioinformatic analysis to predict the functional impact of the remaining variants.

### Familial mutational co-segregation analysis

We confirmed two unreported and one known pathogenic mutation of *LRP6* (NM_002336.3) and excluded other candidate genes. Familial mutational co-segregation analysis was conducted in attainable nuclear family members by targeted Sanger sequencing to validate the candidate mutations. PCR primers for the Sanger sequencing were designed using Primer-BLAST tools (Supplementary Table 8).

### Conservation analysis

The LRP6 amino acid sequences of different species were obtained from UniProt. Evolutionary conservation analysis of the pathogenic mutations identified in this study was performed using the Multiple Sequence Alignment Server (T-coffee).

### Three-dimensional structural analysis

Homology modeling of the four β-propeller-EGF fragments in human LRP6: LRP6-E1E2 (PDB ID: 5gje.1. A) and LRP6-E3E4 (PDB ID: 6h15.1. A) was obtained from the Protein Data Bank (PDB) for conformational change prediction. The three-dimensional structures of wild-type and mutant LRP6 proteins were visualized using the PyMOL2.3 Molecular Graphics System (DeLano Scientific LLC, San Carlos, CA, USA).

### Analysis of susceptible tooth positions caused by LRP6 mutations

We obtained the detailed tooth positions of congenital missing teeth from 39 patients with defined *LRP6* mutations, including 7 patients in this study, 6 patients in our previous study^19^, and 26 patients from other studies^1,17,18,20–22^. Tooth positions were compiled in the upper and lower arches, combined with the left and right sides, that is, the upper right quadrant, upper left quadrant, lower left quadrant, and lower right quadrant. The number of missing teeth was analyzed statistically to determine the prevalence of tooth agenesis in each position. Moreover, missing tooth numbers were counted separately in four divided quadrants. Then, the average rates of the upper and lower arches, as well as the left and right sides, were compared. Statistical analysis was performed using the *χ*2 test using SPSS 24.0, and Prism 8. Statistical significance was set at *P* < 0.05.

### Reporting summary

Further information on research design is available in the Nature Research Reporting Summary linked to this article.

## Supplementary information

Supplementary information

Reporting summary

## Data Availability

The mutations identified in this study were submitted to the ClinVar database, and the accession codes were VCV001296979, VCV000225150, and VCV001296980. The WES data was submitted to SRA with accession number: PRJNA758560. All the web sources used in this study are as follow: gnomAD database, http://gnomad-sg.org/ OMIM, http://www.omim.org/ HGMD, http://www.hgmd.cf.ac.uk/ac/search.php/ MutationTaster, http://www.mutationtaster.org/ PolyPhen-2, http://genetics.bwh.harvard.edu/pph2/ SIFT, http://sift.jcvi.org/ PROVEAN, http://provean.jcvi.org/index.php/ ClinVar database, https://www.ncbi.nlm.nih.gov/clinvar/ Ensembl, http://www.ensembl.org/ Primer-BLAST tool, https://www.ncbi.nlm.nih.gov/tools/primer-blast/index/ Protein Data Bank, PDB, http://www.wwpdb.org/ T-coffee, http://tcoffee.crg.cat/ UniProt, https://www.uniprot.org/

## Code availability

No previously unreported computer code or algorithm was used.
