# Supplementary material for: Rare phenotype: Hand preaxial polydactyly associated with LRP6-related tooth agenesis in humans
Source: NPJ Genom Med. 2021 Nov 10;6:93. doi: 10.1038/s41525-021-00262-0 (PMC8581002; doi:10.1038/s41525-021-00262-0)
Supplement: Supplementary file 1 — Supplementary information [file 41525_2021_262_MOESM1_ESM.pdf]

Supplementary information

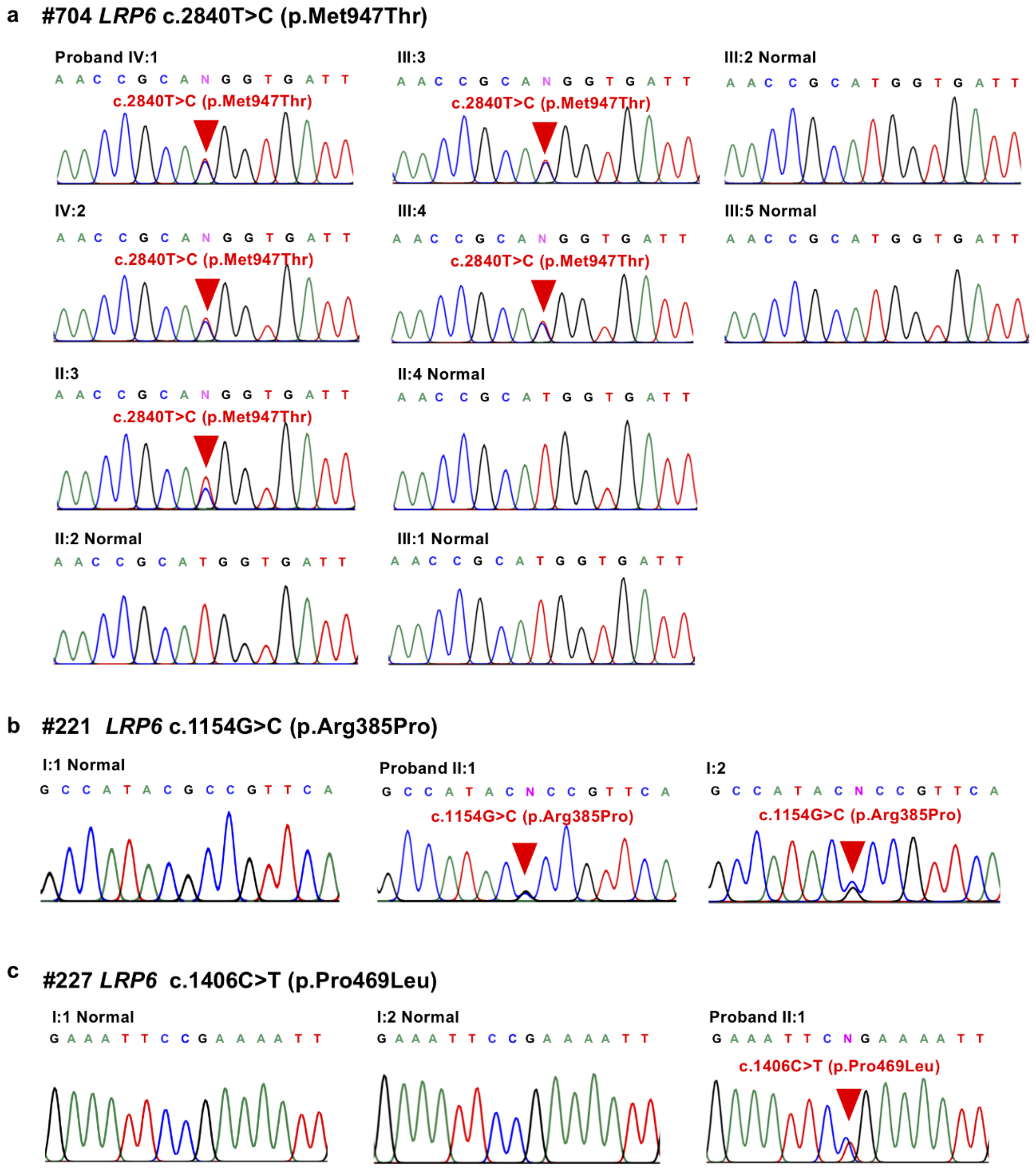

Supplementary Fig. 1. DNA sequencing chromatograms of three heterozygous *LRP6* mutations. (a) A heterozygous *LRP6* missense mutation (c.2840T>C; p.Met947Thr) was identified in the #704 proband (IV:1), her mother (III:3), her uncle (III:4), her grandfather (II:3) and her cousin (IV:2). (b) A heterozygous *LRP6* missense mutation (c.1154G>C; p.Arg385Pro) was identified in the #221 proband (II:1) and her mother (I:2). (c) A heterozygous *LRP6* missense mutation (c.1406C>T; p.Pro469Leu) was identified in the #227 proband (II:1). Red arrowheads indicate the mutation sites.

Supplementary Table 1. Schematic of tooth agenesis in patients harboring *LRP6* mutations in this study and other references.

No.	Gender/Age	Mutation	Mutation Type		Right quadrants							Left quadrants							Missing Number	Proband
				Max	7	6	5	4	3	2	1	1	2	3	4	5	6	7		
				Mand	7	6	5	4	3	2	1	1	2	3	4	5	6	7		
1	Female	c.2840T>C (p.Met947Thr)	Missense		■	□	■	■	■	■	□	□	■	■	■	■	□	□	15	#704/IV:1
					□	□	■	■	□	■	□	□	■	□	■	■	□	□		
2	Female	c.2840T>C (p.Met947Thr)	Missense		□	□	■	■	□	■	□	□	□	■	□	■	□	□	4	#704/III:3
					□	□	□	□	□	□	□	□	□	□	□	□	□	□		
3	Male	c.2840T>C (p.Met947Thr)	Missense		□	□	□	□	□	■	■	□	□	■	□	□	□	□	6	#704/ III:4
					□	□	■	□	■	□	□	□	□	□	□	■	□	□		
4	Female	c.2840T>C (p.Met947Thr)	Missense		□	□	□	□	□	■	□	□	■	□	□	□	□	□	2	#704/ IV:2
					□	□	□	□	□	□	□	□	□	□	□	□	□	□		
5	Male	c.1154G>C (p.Arg385Pro)	Missense		□	□	■	■	■	■	□	□	■	■	■	■	□	□	16	#221/II:1
					□	□	■	■	■	□	■	■	□	■	■	■	□	□		
6	Female	c.1154G>C (p.Arg385Pro)	Missense		□	□	■	■	■	□	□	□	□	■	□	■	□	□	9	#221/I:2
					□	□	■	■	□	□	□	□	□	□	■	■	□	□		
7	Female	c.1406C>T (p.Pro469Leu)	Missense		□	□	□	□	■	■	□	□	■	■	■	□	□	□	11	#227/II:1
					□	□	■	□	□	■	□	■	■	□	■	■	□	□		
8	Male	c.2292G>A (p.Trp764*)	Nonsense		■	■	■	■	□	■	□	□	■	□	■	■	■	■	18	Yu et al., 2021[1]
					□	□	■	■	□	■	■	■	■	□	□	■	□	□		
9	Male	c.2292G>A (p.Trp764*)	Nonsense		□	□	□	□	■	□	□	□	■	□	□	□	□	□	2	Yu et al., 2021[1]
					□	□	□	□	□	□	□	□	□	□	□	□	□	□		
10	Female	c.1095dup (p.Asp366Argfs*13)	Frameshift		■	□	■	■	■	■	□	□	■	■	□	■	□	■	15	Yu et al., 2021[1]
					■	□	■	□	■	□	□	□	■	□	■	■	□	□		
11	Female	c.1095dup (p.Asp366Argfs*13)	Frameshift		□	□	□	■	□	■	□	□	■	■	■	■	□	□	9	Yu et al., 2021[1]
					■	■	□	□	□	□	□	□	□	□	□	□	□	■		

Supplementary Table 1. Continued

No.	Gender/Age	Mutation	Mutation Type		Right quadrants							Left quadrants							Missing Number	Proband
				Max	7	6	5	4	3	2	1	1	2	3	4	5	6	7		
				Mand	7	6	5	4	3	2	1	1	2	3	4	5	6	7		
12	Female	c.1681C>T (p.Arg561*)	Nonsense		□	□	■	■	■	□	□	□	□	■	■	■	□	□	9	Yu et al., 2021[1]
					□	□	■	□	□	□	□	□	□	□	□	■	□	■		
13	Male	c.195dup (p.Tyr66Ilefs*4)	Frameshift		■	□	■	□	■	■	□	□	■	■	□	■	□	■	16	Yu et al., 2021[1]
					■	□	■	□	□	■	■	■	■	□	□	■	□	■		
14	Male	c.56C>T (p.Ala19Val)	Missense		□	□	■	□	□	■	□	□	■	□	■	■	□	□	6	Massink et al., 2015[2]
					□	□	■	□	□	□	□	□	□	□	□	□	□	□		
15	Male	c.56C>T (p.Ala19Val)	Missense		□	□	■	□	□	■	□	□	■	□	□	■	□	□	4	Massink et al., 2015[2]
					□	□	□	□	□	□	□	□	□	□	□	□	□	□		
16	Male	c.56C>T (p.Ala19Val)	Missense		□	□	■	□	■	■	□	□	■	■	□	■	□	□	13	Massink et al., 2015[2]
					■	□	■	□	□	□	■	□	■	■	□	■	□	■		
17	Male	c.56C>T (p.Ala19Val)	Missense		□	□	■	□	□	■	□	□	■	□	□	□	□	□	7	Massink et al., 2015[2]
					□	□	■	□	■	□	□	□	□	■	□	■	□	□		
18	Female	c.1779dupT (p.Glu594*)	Nonsense		□	□	□	□	■	■	□	□	■	■	□	□	□	□	6	Massink et al., 2015[2]
					□	□	■	□	□	□	□	□	□	□	■	□	□	□		
19	Female	c.1779dupT (p.Glu594*)	Nonsense		■	□	■	■	■	■	□	□	■	■	■	■	□	■	20	Massink et al., 2015[2]
					■	□	■	□	■	■	■	■	■	■	■	■	□	□		
20	Male	c.2224_2225dupTT (p.Leu742Phefs*7)	Frameshift		□	□	□	□	■	■	□	□	■	■	■	■	□	□	13	Massink et al., 2015[2]
					□	□	■	■	□	■	■	■	■	□	□	■	□	□		
21	Male	c.2224_2225dupTT (p.Leu742Phefs*7)	Frameshift		■	□	■	■	■	■	□	□	■	■	□	□	□	□	16	Massink et al., 2015[2]
					■	□	■	■	□	□	■	■	■	□	■	■	□	■		
22	Female	c.2224_2225dupTT (p.Leu742Phefs*7)	Frameshift		■	□	■	■	■	■	□	□	■	■	■	■	□	□	20	Massink et al., 2015[2]
					■	□	■	■	■	■	■	■	■	□	■	■	□	■		
23	Male	c.1144_1145dupAG (p.Ala383Glyfs*8)	Frameshift		□	□	■	■	□	■	□	□	■	□	■	□	□	□	12	Massink et al., 2015[2]
					□	□	■	■	□	■	■	■	■	□	□	■	□	□		

Supplementary Table 1. Continued

No.	Gender/Age	Mutation	Mutation Type		Right quadrants							Left quadrants							Missing Number	Proband
				Max	7	6	5	4	3	2	1	1	2	3	4	5	6	7		
				Mand	7	6	5	4	3	2	1	1	2	3	4	5	6	7		
24	Male	c.4594delG (p.Cys1532Alafs*16)	Frameshift		□	□	■	■	■	■	□	□	■	■	■	■	□	□	17	Ockeloen et al., 2016[3]
					□	□	■	■	□	■	■	■	■	■	■	■	□	□		
25	Female	c.4594delG (p.Cys1532Alafs*16)	Frameshift		□	□	■	■	□	■	□	□	■	□	■	■	□	□	14	Ockeloen et al., 2016[3]
					□	□	■	■	□	■	■	■	■	□	■	■	□	□		
26	Male	c.3398-2A>C	Splicing		■	□	■	■	□	■	□	□	■	□	□	■	□	■	9	Ockeloen et al., 2016[3]
					□	□	■	□	□	□	□	□	□	□	□	■	□	□		
27	Female	c.517C>G (p.Arg173Gly)	Missense		□	□	■	□	■	■	□	□	■	■	■	■	□	□	9	Ockeloen et al., 2016[3]
					□	□	■	□	□	□	□	□	□	□	□	■	□	□		
28	Male	c.1406C>T (p.Pro469Leu)	Missense		□	□	□	□	■	■	□	□	■	■	■	□	□	□	7	Ockeloen et al., 2016[3]
					□	□	□	■	□	■	□	□	□	□	□	□	□	□		
29	Male	c.1609G>A (p.Gly537Arg)	Missense		■	□	■	■	□	■	□	□	■	□	□	■	■	□	10	Ockeloen et al., 2016[3]
					□	□	■	□	□	□	□	□	□	□	■	■	□	□		
30	Female	c.1609G>A (p.Gly537Arg)	Missense		□	□	□	□	□	■	□	□	■	□	□	□	□	□	2	Ockeloen et al., 2016[3]
					□	□	□	□	□	□	□	□	□	□	□	□	□	□		
31	Male	c.2994+1G>A	Splicing		□	□	■	■	■	■	□	□	■	■	■	■	□	□	13	Ockeloen et al., 2016[3]
					□	□	■	■	■	□	□	□	□	□	■	■	□	□		
32	Male	c.4082-2A>G	Splicing		□	□	□	□	□	■	□	□	■	□	□	□	□	■	6	Ockeloen et al., 2016[3]
					□	□	■	□	□	□	□	□	□	□	□	■	□	■		
33	Female	c.3607+3-6 delAAGT	Splicing		□	□	■	■	□	■	□	□	■	□	■	■	□	□	16	Dinckan et al., 2018[4]
					□	□	■	■	■	■	■	■	■	■	■	■	□	□		
34	Male	Interstitial loss of 290 kb in 12p13.2	Copy number variant		□	□	□	□	■	■	□	□	■	■	□	□	□	□	9	Ross et al., 2019[5]
					□	□	□	□	■	■	■	■	■	□	□	□	□	□		

Supplementary Table 1. Continued

No.	Gender/Age	Mutation	Mutation Type		Right quadrants							Left quadrants							Missing Number	Proband
				Max	7	6	5	4	3	2	1	1	2	3	4	5	6	7		
				Mand	7	6	5	4	3	2	1	1	2	3	4	5	6	7		
35	Male	Interstitial loss of 290 kb in 12p13.2	Copy number variant		□	□	□	□	■	■	□	□	■	■	□	□	□	□	10	Ross et al., 2019[5]
					□	□	□	□	■	■	■	■	■	■	□	□	□	□		
36	Male	Interstitial loss of 290 kb in 12p13.2	Copy number variant		□	□	□	□	■	■	□	□	■	■	□	□	□	□	10	Ross et al., 2019[5]
					□	□	□	□	■	■	■	■	■	■	□	□	□	□		
37	Female	c. 711G>T p.Leu237Phe	Missense		■	□	■	■	□	■	□	□	■	□	■	■	□	■	17	Wang et al., 2021[6]
					■	□	■	■	□	□	■	■	■	□	■	■	□	■		
38	Female	c. 711G>T p.Leu237Phe	Missense		□	□	□	□	□	■	□	□	□	□	□	□	□	□	1	Wang et al., 2021[6]
					□	□	□	□	□	□	□	□	□	□	□	□	□	□		
39	Female	c. 711G>T p.Leu237Phe	Missense		□	□	□	□	□	□	□	□	■	□	□	□	□	□	1	Wang et al., 2021[6]
					□	□	□	□	□	□	□	□	□	□	□	□	□	□		

NOTE: Missing teeth are marked with black blocks. Reference sequence: NM_002336.3 and NP_002327.

Supplementary References:

1. Yu, M. *et al.* Lrp6 Dynamic Expression in Tooth Development and Mutations in Oligodontia. *J Dent Res* 100, 415-422 (2021).

2. Massink, M.P. *et al.* Loss-of-Function Mutations in the WNT Co-receptor LRP6 Cause Autosomal-Dominant Oligodontia. *Am J Hum Genet* 97, 621-6 (2015).

3. Ockeloen, C.W. *et al.* Novel mutations in LRP6 highlight the role of WNT signaling in tooth agenesis. *Genet Med* 18, 1158-1162 (2016).

4. Dinckan, N. *et al.* Whole-Exome Sequencing Identifies Novel Variants for Tooth Agenesis. *J Dent Res* 97, 49-59 (2018).

5. Ross, J. *et al.* Concurrent manifestation of oligodontia and thrombocytopenia caused by a contiguous gene deletion in 12p13.2: A three-generation clinical report. *Mol Genet Genomic Med* 7, e679 (2019).

6. Wang, H. et al. A novel missense mutation of *LRP6* identified by whole-exome sequencing in a Chinese family with non-syndromic tooth agenesis. Orthod Craniofac Res 24, 233-240 (2021).

Supplementary Table 2. Polydactyly/syndactyly-related genes screening results of proband (#704 IV:1) by WES.

No.	*Gene Name*	Location	Mutation	Function	ID	Priority	gnomAD	SIFT	Polyphen-2	Mutation Taster	Functional Effect
1	*DHCR7* NM_001163817	11q13.4	c. 1272C>T p.Gly424Gly	synonymous	rs909217	Low	0.5601	-	-	-	Benign
			c. 1158T>C p.Asp386Asp	synonymous	rs760241	Low	0.8528	Damging		Polymorphism	Benign
			c.438T>C p.Asn146Asn	synonymous	rs949177	Low	0.8711	-	-	-	Benign
			c.231C>T p.Thr77Thr	synonymous	rs4316537	Low	0.078	-	-	-	Benign
			c.207T>C p.Thr69Thr	synonymous	rs1790334	Low	0.872	-	-	-	Benign
			c.189G>A p.Gln63Gln	synonymous	rs1044482	Low	0.5649	-	-	-	Benign
2	*FBLN1* NM_001996	22q13.31	c. 422A>G p.Gln141Arg	nonsynonymous	rs136730	Low	0.9986	Tolerant	Benign	Polymorphism	Benign
3	*FGFR2* NM_001144915	10q26.12	c.2107C>T p.Leu703Leu	synonymous	rs1047057	Low	0.5559	-	-	-	Benign
			c.351A>G p.Val117Val	synonymous	rs1047100	Low	0.7813				Benign
			c.17G>C p.Arg6Pro	nonsynonymous	rs3750819	Low	0.0076	Tolerant	Benign	-	Benign
4	*FGF16*	Xq21.1	-	-	-	-	-	-	-	-	-

Supplementary Table 2. Continued

No.	*Gene Name*	Location	Mutation	Function	ID	Priority	gnomAD	SIFT	Polyphen-2	Mutation Taster	Functional Effect
5	*FMN1* NM_001103184	15q13.3	c.3387T>C p.Phe1129Phe	synonymous	rs7162695	Low	0.1399				Benign
			c.2001C>T p.Pro667Pro	synonymous	rs2930131	Low	0.5988				Benign
			c.512G>T p.Gly171Val	nonsynonymous	rs11858145	Low	0.7006	Tolerant	Benign		Benign
			c.772A>G p.Lys258Glu	nonsynonymous	rs61744870	Low	0.0062	Tolerant	Benign		Likely Benign
6	*HOXD13* NM_000523	2q31.1	c.204G>A p.Ala68Ala	synonymous		Low	0.343				Benign
			c.375G>A p.Leu125Leu	synonymous		Low	0.3145				Benign
7	*GLI3* NM_000168	7p14.1	c.547A>G p.Thr183Ala	nonsynonymous	rs846266	Low	0.5439	Tolerant	Benign	Polymorphism	Benign
8	*GLI1* NM_001160045	12q13.3	c.192G>A p.Glu64Glu	synonymous	rs2228225	Low	0.4972	-	-	-	Benign
			c.2414G>A p.Gly805Asp	nonsynonymous	rs2228224	Low	0.4985	Damaging	Benign	Polymorphism	Benign
			c.2914G>C p.Glu972Gln	nonsynonymous	rs2228226	Low	0.6015	Tolerant	Benign	Polymorphism	Benign
9	*GREM1*	-	-	-	-	-	-	-	-	-	-
10	*GJA1*	-	-	-	-	-	-	-	-	-	-
11	*LMBR1*	-	-	-	-	-	-	-	-	-	-

Supplementary Table 2. Continued

No.	*Gene Name*	Location	Mutation	Function	ID	Priority	gnomAD	SIFT	Polyphen-2	Mutation Taster	Functional Effect
12	*LRP4* NM_002334	11p11.2	c.4937G>A p.Arg1646Gln	nonsynonymous	rs3816614	Low	0.6804	Tolerant	Benign	Polymorphism	Benign
			c.4905C>T p.Phe1635Phe	synonymous	rs2290883	Low	0.2086	-	-	Polymorphism	Benign
			c.3608C>T p.Ala1203Val	nonsynonymous	rs2306033	Low	0.2083	Tolerant	Benign	Polymorphism	Benign
			c.3256A>G p.Ile1086Val	nonsynonymous	rs6485702	Low	0.5881	Tolerant	Benign		Benign
			c.1638A>G p.Lys546Lys	synonymous	rs10838631	Low	0.9888	-	-	-	Benign
			c.1623T>C p.Asp541Asp	synonymous	rs10769215	Low	0.9991	-	-	-	Benign
13	*MIPOLI*	-	-	-	-	-	-	-	-	-	-
14	*MKKS*	-	-	-	-	-	-	-	-	-	-
15	*PAPA2*	-	-	-	-	-	-	-	-	-	-
16	*PAPA3*	-	-	-	-	-	-	-	-	-	-
17	*PAPA4*	-	-	-	-	-	-	-	-	-	-
18	*PAPA5*	-	-	-	-	-	-	-	-	-	-
19	*PITX1* NM_002653	5q31.1	c.G896C p.Gly299Ala	nonsynonymous	rs479632	Low	0.2919	Tolerant	Benign	Polymorphism	Benign
20	*PPD3*	-	-	-	-	-	-	-	-	-	-
21	*RAB23*	-	-	-	-	-	-	-	-	-	-
22	*SHH*	-	-	-	-	-	-	-	-	-	-
23	*SHFM2*	-	-	-	-	-	-	-	-	-	-
24	*TWIST1*	-	-	-	-	-	-	-	-	-	-
25	*ZNF141*	-	-	-	-	-	-	-	-	-	-

Supplementary Table 3. Polydactyly/syndactyly-related genes screening results of proband's mother (#704 III:3) by WES.

No.	*Gene Name*	Location	Mutation	Function	ID	Priority	gnomAD	SIFT	Polyphen-2	Mutation Taster	Functional Effect
1	*DHCR7* NM_001163817	11q13.4	c.1272C>T p.Gly424Gly	synonymous	rs909217	Low	0.5601	-	-	-	Benign
			c.1158T>C p.Asp386Asp	synonymous	rs760241	Low	0.8528	Damging	-	Polymorphism	Benign
			c.438T>C p.Asn146Asn	synonymous	rs949177	Low	0.8711	-	-	-	Benign
			c.207T>C p.Thr69Thr	synonymous	rs1790334	Low	0.872	-	-	-	Benign
			c.189G>A p.Gln63Gln	synonymous	rs1044482	Low	0.5649	-	-	-	Benign
2	*FBLN1* NM_001996	22q13.31	c.422A>G p.Gln141Arg	nonsynonymous	rs136730	-	0.9986	Tolerant	Benign	Polymorphism	Benign
3	*FGFR2* NM_001144915	10q26.12	c.2107C>T p.Leu703Leu	synonymous	rs1047057	Low	0.5559				Benign
			c.351A>G p.Val117Val	synonymous	rs1047100	Low	0.7813				Benign
4	*FGF16*	Xq21.1	-	-	-	-	-	-	-	-	-
5	*FMN1* NM_001103184	15q13.3	c.3387T>C p.Phe1129Phe	synonymous	rs7162695	Low	0.1399	-	-	-	Benign
			c.2001C>T p.Pro667Pro	synonymous	rs2930131	Low	0.5988	-	-	-	Benign
			c.512G>T p.Gly171Val	nonsynonymous	rs11858145	Low	0.7006	Tolerant	Benign	-	Benign
6	*HOXD13* NM_000523	2q31.1	c.375G>A p.Leu125Leu	synonymous	rs847151	Low	0.3145	-	-	-	Benign

Supplementary Table 3. Continued

No.	*Gene Name*	Location	Mutation	Function	ID	Priority	gnomAD	SIFT	Polyphen-2	Mutation Taster	Functional Effect
7	*GLI3* NM_000168	7p14.1	c.2993C>T p.Pro998Leu	nonsynonymous	rs929387	Low	0.4251	Tolerant	Benign	polymorphism	Benign
			c.547A>G p.Thr183Ala	nonsynonymous	rs846266	Low	0.5439	Tolerant	Benign	Polymorphism	Benign
8	*GLI1*	12q13.3	-	-	-	-	-	-	-	-	-
9	*GREM1*	-	-	-	-	-	-	-	-	-	-
10	*GJA1*	-	-	-	-	-	-	-	-	-	-
11	*LMBR1*	-	-	-	-	-	-	-	-	-	-
12	*LRP4* NM_002334	11p11.2	c.4937G>A p.Arg1646Gln	nonsynonymous	rs3816614	Low	0.6804	Tolerant	Benign	Polymorphism	Benign
			c.3256A>G p.Ile1086Val	nonsynonymous	rs6485702	Low	0.5881	Tolerant	Benign	Polymorphism	Benign
			c.1638A>G p.Lys546Lys	synonymous	rs10838631	Low	0.9888	-	-	-	Benign
			c.1623T>C p.Asp541Asp	synonymous	rs10769215	Low	0.9991	-	-	-	Benign
			c.997G>A p.Gly333Arg	nonsynonymous	rs61744209	Low	0.0032	Damaging	Deleterious	Disease Causing	Benign
13	*MIPOLI*	-	-	-	-	-	-	-	-	-	-
14	*MKKS*	-	-	-	-	-	-	-	-	-	-
15	*PAPA2*	-	-	-	-	-	-	-	-	-	-
16	*PAPA3*	-	-	-	-	-	-	-	-	-	-
17	*PAPA4*	-	-	-	-	-	-	-	-	-	-
18	*PAPA5*	-	-	-	-	-	-	-	-	-	-

Supplementary Table 3. Continued

No.	*Gene Name*	Location	Mutation	Function	ID	Priority	gnomAD	SIFT	Polyphen-2	Mutation Taster	Functional Effect
19	*PITX1* NM_002653	5q31.1	c.896G>C p.Gly299Ala	nonsynonymous	rs479632	Low	0.2919	Tolerant	Benign	Polymorphism	Benign
20	*PPD3*	-	-	-	-	-	-	-	-	-	-
21	*RAB23*	-	-	-	-	-	-	-	-	-	-
22	*SHH* NM_000193	7q36.3	c.869G>A p.Gly290Asp	nonsynonymous	rs104894047	Low	0.0033	Tolerant	Probably Damaging	-	Benign
23	*SHFM2*	-	-	-	-	-	-	-	-	-	-
24	*TWIST1*	7p21.1	-	-	-	-	-	-	-	-	-
25	*ZNF141*	-	-	-	-	-	-	-	-	-	-

Supplementary Table 4. Polydactyly/syndactyly-related genes screening results of proband's father (#704 III:2) by WES.

No.	*Gene Name*	Location	Mutation	Function	ID	Priority	gnomAD	SIFT	Polyphen-2	Mutation Taster	Function Effect
1	*DHCR7* NM_001163817	11q13.4	c.1272C>T p.Gly424Gly	synonymous	rs909217	Low	0.5601	-	-	-	Benign
			c.1158T>C p.Asp386Asp	synonymous	rs760241	Low	0.8528	Damaging	-	Polymorphism	Benign
			c.438T>C p.Asn146Asn	synonymous	rs949177	Low	0.8711	-	-	-	Benign
			c.231C>T p.Thr77Thr	synonymous	rs4316537	Low	0.078	-	-	-	Benign
			c.207T>C p.Thr69Thr	synonymous	rs1790334	Low	0.872	-	-	-	Benign
			c.189G>A p.Gln63Gln	synonymous	rs1044482	Low	0.5649	-	-	-	Benign
2	*FBLN1* NM_001996	22q13.31	c.422A>G p.Gln141Arg	synonymous	rs136730	Low	0.9986	Tolerant	Benign	Polymorphism	Benign
3	*FGFR2* NM_001144915	10q26.12	c.17G>C p.Arg6Pro	nonsynonymous	rs3750819	High	0.0076	Tolerant	Benign	-	Benign
			c.2107C>T p.Leu703Leu	synonymous	rs1047057	Low	0.5559	-	-	-	Benign
			c.351A>G p.Val117Val	synonymous	rs1047100	Low	0.7813	-	-	-	Benign
4	*FGF16*	Xq21.1	-	-	-	-	-	-	-	-	-

Supplementary Table 4. Continued

No.	*Gene Name*	Location	Mutation	Function	ID	Priority	gnomAD	SIFT	Polyphen-2	Mutation Taster	Function Effect
5	*FMN1* NM_001103184	15q13.3	c.2048C>T p.Pro683Leu	nonsynonymous	rs117804335	Low	0.0473	Tolerant	Benign	-	Benign
			c.2001C>T p.Pro667Pro	synonymous	rs2930131	Low	0.5988	-	-	-	Benign
			c.512G>T p.Gly171Val	nonsynonymous	rs11858145	Low	0.7006	Tolerant	Benign	-	Benign
			c.858C>T p.Ser286Ser	synonymous	rs28377066	Low	0.4242	-	-	-	Benign
			c.805C>G p.Pro269Ala	nonsynonymous	rs28507600	Low	0.4246	Tolerant	Benign	-	Benign
			c.772A>G p.Lys258Glu	nonsynonymous	rs61744870	Low	0.0062	Tolerant	Benign	-	Benign
6	*HOXD13* NM_000523	2q31.1	c.204G>A p.Ala68Ala	synonymous	rs2518053	Low	0.343	-	-	-	Benign
7	*GLI3* NM_000168	7p14.1	c.2993C>T p.Pro998Leu	nonsynonymous	rs929387	Low	0.4251	Tolerant	Benign	Polymorphism	Benign
			c.547A>G p.Thr183Ala	nonsynonymous	rs846266	Low	0.5439	Tolerant	Benign	Polymorphism	Benign
8	*GLI1* NM_001160045	12q13.3	c.192G>A p.Glu64Glu	synonymous	rs2228225	Low	0.4972	-	-	-	Benign
			c.2414G>A p.Gly805Asp	nonsynonymous	rs2228224	Low	0.4985	Damaging	Benign	Polymorphism	Benign
			c.2914G>C p.Glu972Gln	nonsynonymous	rs2228226	Low	0.6015	Tolerant	Benign	Polymorphism	Benign
9	*GREM1*	-	-	-	-	-	-	-	-	-	-
10	*GJA1*	-	-	-	-	-	-	-	-	-	-

Supplementary Table 4. Continued

No.	*Gene Name*	Location	Mutation	Function	ID	Priority	gnomAD	SIFT	Poly phen-2	Mutation Taster	Function Effect
11	*LMBR1*	-	-	-	-	-	-	-	-	-	-
12	*LRP4* NM_002334	11p11.2	c.4905C>T p.Phe1635Phe	synonymous	rs2290883	Low	0.2086	-	-	-	-
			c.3608C>T p.Ala1203Val	nonsynonymous	rs2306033	Low	0.2083	Tolerant	Benign	Polymorphism	Benign
			c.1638A>G p.Lys546Lys	synonymous	rs10838631	Low	0.9888	-	-	-	Benign
			c.1623T>C p.Asp541Asp	synonymous	rs10769215	Low	0.9991	-	-	-	Benign
13	*MIPOLI*	-	-	-	-	-	-	-	-	-	-
14	*MKKS*	-	-	-	-	-	-	-	-	-	-
15	*PAPA2*	-	-	-	-	-	-	-	-	-	-
16	*PAPA3*	-	-	-	-	-	-	-	-	-	-
17	*PAPA4*	-	-	-	-	-	-	-	-	-	-
18	*PAPA5*	-	-	-	-	-	-	-	-	-	-
19	*PITX1* NM_002653	5q31.1	c.896G>C p.Gly299Ala	nonsynonymous	rs479632	Low	0.2919	Tolerant	Benign	Polymorphism	Benign
20	*PPD3*	-	-	-	-	-	-	-	-	-	-
21	*RAB23*	-	-	-	-	-	-	-	-	-	-
22	*SHH*	-	-	-	-	-	-	-	-	-	-
23	*SHFM2*	-	-	-	-	-	-	-	-	-	-
24	*TWIST1* NM_000474	7p21.1	c.259_276del p.Ala87_Gly92del	nonframeshift deletion	rs544465774	Medium	0.009	-	-	-	-
25	*ZNF141*	-	-	-	-	-	-	-	-	-	-

Supplementary Table 5 Pair test of missing teeth number in the same position of maxillary and mandibular.

	Tooth position							
	**CI**	**LI**	**CA**	**PM1**	**PM2**	**M1**	**M2**	**Total**
p	<0.0001	<0.0001	<0.0001	0.2566	0.4041	0.6201	>0.9999	0.0327

NOTE: Max, maxillary; Mand, mandibular; CI, central incisor; LI, lateral incisor; CA, canine; PM1, first premolar; PM2, second premolar; M1, first molar; M2, second molar.

Supplementary Table 6 Pair test of missing teeth number in the same position of left and right quadrant.

		Tooth position							
		**CI**	**LI**	**CA**	**PM1**	**PM2**	**M1**	**M2**	**Total**
p	Max	>0.9999	>0.9999	0.8196	>0.9999	>0.9999	>0.9999	0.5843	0.7932
	Mand	>0.9999	0.6470	0.5986	>0.9999	>0.9999	>0.9999	>0.9999	>0.9999

NOTE: Max, maxillary; Mand, mandibular; CI, central incisor; LI, lateral incisor; CA, canine; PM1, first premolar; PM2, second premolar; M1, first molar; M2, second molar.

Supplementary Table 7. *LRP6* mutations and detailed phenotypes identified in the literature and this study.

No.	DNA change	Nucleotide change type	Amino acid change	Mutation type	Location	Zygosity	Phenotype	Reference
1	c.1154G>C	Substitution	p.Arg385Pro	Missense	Exon 6	Heterozygous	Oligodontia	This study
2	c.2840T>C	Substitution	p.Met947Thr	Missense	Exon 13	Heterozygous	Oligodontia, Hypodontia, Polysyndactyly	This study
3	c.1681C>T	Substitution	p.Arg561*	Nonsense	Exon 8	Heterozygous	Oligodontia	Yu et al., 2021[1]
4	c.2292G>A	Substitution	p.Trp764*	Nonsense	Exon 11	Heterozygous	Hypohidrotic, Ectodermal dysplasia	Yu et al., 2021[1]
5	c.195dup	Insertion	p.Tyr66Ilefs*4	Frameshift	Exon 2	Heterozygous	Oligodontia	Yu et al., 2021[1]
6	c.1095dup	Insertion	p.Asp366Argfs*13	Frameshift	Exon 6	Heterozygous	Oligodontia	Yu et al., 2021[1]
7	c.56C>T	Substitution	p.Ala19Val	Missense	Exon 2	Heterozygous	Oligodontia	Massink et al., 2015[2]
8	c.1779dupT	Insertion	p.Glu594*	Nonsense	Exon 9	Heterozygous	Oligodontia	Massink et al., 2015[2]
9	c.1144_1145dupAG	Insertion	p.Ala383Glyfs*8	Frameshift	Exon 6	Heterozygous	Oligodontia	Massink et al., 2015[2]
10	c.2224_2225dupTT	Insertion	p.Leu742Phefs*7	Frameshift	Exon 10	Heterozygous	Oligodontia	Massink et al., 2015[2]
11	c.517C>G	Substitution	p.Arg173Gly	Missense	Exon 3	Heterozygous	Oligodontia	Ockeloen et al., 2016[3]
12	c.1406C>T	Substitution	p.Pro469Leu	Missense	Exon 7	Heterozygous	Oligodontia	This study; Ockeloen et al., 2016[3]
13	c.1609G>A	Substitution	p.Gly537Arg	Missense	Exon 8	Heterozygous	Oligodontia	Ockeloen et al., 2016[3]
14	c.4594delG	Deletion	p.Cys1532Alafs*16	Frameshift	Exon 23	Heterozygous	Oligodontia, Bilateral cleft lip and palate	Ockeloen et al., 2016[3]

Supplementary Table 7. **Continued**

No.	DNA change	Nucleotide change type	Amino acid change	Mutation type	Location	Zygosity	Phenotype	Reference
15	c.2994+1G>A	Substitution	p.?	Splicing	Intron 13	Heterozygous	Oligodontia	Ockeloen et al., 2016[3]
16	c.3398-2A>C	Substitution	p.?	Splicing	Intron 15	Heterozygous	Oligodontia	Ockeloen et al., 2016[3]
17	c.4082-2A>G	Substitution	p.?	Splicing	Intron 19	Heterozygous	Oligodontia	Ockeloen et al., 2016[3]
18	c.3373C>T	Substitution	p.Arg1125*	Nonsense	Exon 15	Heterozygous	Hypodontia, Bilateral cleft lip and palate	Basha et al., 2018[4]
19	c.3607+3_6delAAGT	Deletion	p.?	Splicing	Intron 16	Heterozygous	Oligodontia	Dinckan et al., 2018[5]
20	Interstitial loss of 290 kb in 12p13.2		–	Copy number variant	Exons and introns 16-23	Heterozygous	Oligodontia, Thrombocytopenia	Ross et al., 2019[6]
21	c. 711G>T	Substitution	p.Leu237Phe	Missense	Exon 4	Heterozygous	Oligodontia	Wang et al., 2021[7]

Supplementary References：

1. Yu, M. *et al.* Lrp6 Dynamic Expression in Tooth Development and Mutations in Oligodontia. *J Dent Res* 100, 415-422 (2021).

2. Massink, M.P. *et al.* Loss-of-Function Mutations in the WNT Co-receptor LRP6 Cause Autosomal-Dominant Oligodontia. *Am J Hum Genet* 97, 621-6 (2015).

3. Ockeloen, C.W. *et al.* Novel mutations in LRP6 highlight the role of WNT signaling in tooth agenesis. *Genet Med* 18, 1158-1162 (2016).

4. Basha, M. *et al.* Whole exome sequencing identifies mutations in 10% of patients with familial non-syndromic cleft lip and/or palate in genes mutated in well-known syndromes. *J Med Genet* 55, 449-458 (2018).

5. Dinckan, N. *et al.* Whole-Exome Sequencing Identifies Novel Variants for Tooth Agenesis. *J Dent Res* 97, 49-59 (2018).

6. Ross, J. *et al.* Concurrent manifestation of oligodontia and thrombocytopenia caused by a contiguous gene deletion in 12p13.2: A three-generation clinical report. *Mol Genet Genomic Med* 7, e679 (2019).

7. Wang, H. et al. A novel missense mutation of *LRP6* identified by whole-exome sequencing in a Chinese family with non-syndromic tooth agenesis. Orthod Craniofac Res 24, 233-240 (2021).

Supplementary Table 8. Primer information of the involved coding exons for the *LRP6* amplification.

Exon	Forward primer（5'-3'）	Reverse primer（5'-3'）
6	GAGAGTGCACATCCTTTTGTGT	AGAGCTGTTTACTGGAAACCAA
7	GGAGGCATTTTCAGGATTGGC	GGCACCCCAGACAACTGTTA
13	GCCACCCACCAGACAGAATA	GTGAAACAGAGTGGTTGGTGAG
