# Supplementary material for: Rare phenotype: Hand preaxial polydactyly associated with LRP6-related tooth agenesis in humans
Source: NPJ Genom Med. 2021 Nov 10;6:93. doi: 10.1038/s41525-021-00262-0 (PMC8581002; doi:10.1038/s41525-021-00262-0)
Supplement: Supplementary file 2 — Reporting summary [file 41525_2021_262_MOESM2_ESM.pdf]

nature portfolio

Corresponding author(s): Dong Han

Last updated by author(s): Sep 3, 2021

Reporting Summary

Nature Portfolio wishes to improve the reproducibility of the work that we publish. This form provides structure for consistency and transparency in reporting. For further information on Nature Portfolio policies, see our Editorial Policies and the Editorial Policy Checklist.

Statistics

For all statistical analyses, confirm that the following items are present in the figure legend, table legend, main text, or Methods section.

n/a	Confirmed	
☐	☒	The exact sample size (n) for each experimental group/condition, given as a discrete number and unit of measurement
☐	☒	A statement on whether measurements were taken from distinct samples or whether the same sample was measured repeatedly
☐	☒	The statistical test(s) used AND whether they are one- or two-sided<br>*Only common tests should be described solely by name; describe more complex techniques in the Methods section.*
☒	☐	A description of all covariates tested
☒	☐	A description of any assumptions or corrections, such as tests of normality and adjustment for multiple comparisons
☒	☐	A full description of the statistical parameters including central tendency (e.g. means) or other basic estimates (e.g. regression coefficient) AND variation (e.g. standard deviation) or associated estimates of uncertainty (e.g. confidence intervals)
☐	☒	For null hypothesis testing, the test statistic (e.g. F, t, r) with confidence intervals, effect sizes, degrees of freedom and P value noted<br>*Give P values as exact values whenever suitable.*
☒	☐	For Bayesian analysis, information on the choice of priors and Markov chain Monte Carlo settings
☒	☐	For hierarchical and complex designs, identification of the appropriate level for tests and full reporting of outcomes
☒	☐	Estimates of effect sizes (e.g. Cohen's d, Pearson's r), indicating how they were calculated

Our web collection on statistics for biologists contains articles on many of the points above.

Software and code

Policy information about availability of computer code

Data collection	gnomAD database, http://gnomad-sg.org/<br>OMIM, http://www.omim.org/<br>HGMD, http://www.hgmd.cf.ac.uk/ac/search.php/<br>MutationTaster, http://www.mutationtaster.org/<br>PolyPhen-2, http://genetics.bwh.harvard.edu/pph2/<br>SIFT, http://sift.jcvi.org/<br>PROVEAN, http://provean.jcvi.org/index.php/<br>ClinVar database, https://www. ncbi.nlm.nih.gov/clinvar/<br>Ensembl, http://www.ensembl.org/<br>Primer-BLAST tool, https://www.ncbi.nlm.nih.gov/tools/primer-blast/index/<br>Protein Data Bank, PDB, http://www.wwpdb.org/<br>T-coffee, http://tcoffee.crg.cat/<br>UniProt, https://www.uniprot.org/<br>No previously unreported computer code or algorithm was used.
Data analysis	Statistical analysis was performed using the Chi-squared χ2 test using SPSS 24.0, and Prism 8. Statistical significance was set at $P < 0.05$.

For manuscripts utilizing custom algorithms or software that are central to the research but not yet described in published literature, software must be made available to editors and reviewers. We strongly encourage code deposition in a community repository (e.g. GitHub). See the Nature Portfolio guidelines for submitting code & software for further information.

Data

Policy information about availability of data

All manuscripts must include a data availability statement. This statement should provide the following information, where applicable:

- Accession codes, unique identifiers, or web links for publicly available datasets
- A description of any restrictions on data availability
- For clinical datasets or third party data, please ensure that the statement adheres to our policy

The mutations identified in this study were submitted to the ClinVar database, and the submission ID was SUB9107758.

Field-specific reporting

Please select the one below that is the best fit for your research. If you are not sure, read the appropriate sections before making your selection.

☒ Life sciences ☐ Behavioural & social sciences ☐ Ecological, evolutionary & environmental sciences

For a reference copy of the document with all sections, see nature.com/documents/nr-reporting-summary-flat.pdf

Life sciences study design

All studies must disclose on these points even when the disclosure is negative.

Sample size	We obtained the detailed tooth positions of congenital missing teeth from 39 patients with defined LRP6 mutations, including 7 patients in this study, 6 patients in our previous study, and 26 patients from other studies.
Data exclusions	We excluded the tooth agenesis patients carrying with other gene mutations, and the unclear missing tooth position of patients with LRP6 mutations.
Replication	This study was only a case report with the prevalence analysis of tooth agenesis in each position,and the clinical trial or cell and molecular biology experiment was not included, so the replication is not involved.
Randomization	The randomization was not used since this study was a case report.
Blinding	The blinding was not used since this study was a case report.

Reporting for specific materials, systems and methods

We require information from authors about some types of materials, experimental systems and methods used in many studies. Here, indicate whether each material, system or method listed is relevant to your study. If you are not sure if a list item applies to your research, read the appropriate section before selecting a response.

Materials & experimental systems

n/a	Involved in the study	
☒	☐	Antibodies
☒	☐	Eukaryotic cell lines
☒	☐	Palaeontology and archaeology
☒	☐	Animals and other organisms
☐	☒	Human research participants
☒	☐	Clinical data
☒	☐	Dual use research of concern

Methods

n/a	Involved in the study	
☒	☐	ChIP-seq
☒	☐	Flow cytometry
☒	☐	MRI-based neuroimaging

Human research participants

Policy information about studies involving human research participants

Population characteristics	#704-IV:1: 16-year-old, female, LRP6 heterozygous missense mutation (c.2840T>C;p.Met947Thr), tooth agenesis.<br>#221-II:1: 31-year-old, male, LRP6 heterozygous missense mutation (c.1154G>C; p.Arg385Pro), tooth agenesis.<br>#227-II:1: 17-year-old, female, LRP6 heterozygous missense mutation (c.1406C>T;p.Pro469Leu), tooth agenesis.
Recruitment	Three unrelated families with inherited tooth agenesis were recruited from the Department of Prosthodontics at the Peking University School and Hospital of Stomatology (Beijing, China).

Ethics oversight	All experiments were approved by the Ethics Committee of Peking University School and Hospital of Stomatology (PKUSSIRB-201736082).

Note that full information on the approval of the study protocol must also be provided in the manuscript.
